# BDNF-Regulated Modulation of Striatal Circuits and Implications for Parkinson’s Disease and Dystonia

**DOI:** 10.3390/biomedicines12081761

**Published:** 2024-08-05

**Authors:** Daniel Wolf, Maurilyn Ayon-Olivas, Michael Sendtner

**Affiliations:** Institute of Clinical Neurobiology, University Hospital Wuerzburg, 97078 Wuerzburg, Germanyayonolivas_m@ukw.de (M.A.-O.)

**Keywords:** brain-derived neurotrophic factor, BDNF, TrkB, neuronal plasticity, dopamine, neurological diseases, Parkinson’s disease, PD, dystonia

## Abstract

Neurotrophins, particularly brain-derived neurotrophic factor (BDNF), act as key regulators of neuronal development, survival, and plasticity. BDNF is necessary for neuronal and functional maintenance in the striatum and the substantia nigra, both structures involved in the pathogenesis of Parkinson’s Disease (PD). Depletion of BDNF leads to striatal degeneration and defects in the dendritic arborization of striatal neurons. Activation of tropomyosin receptor kinase B (TrkB) by BDNF is necessary for the induction of long-term potentiation (LTP), a form of synaptic plasticity, in the hippocampus and striatum. PD is characterized by the degeneration of nigrostriatal neurons and altered striatal plasticity has been implicated in the pathophysiology of PD motor symptoms, leading to imbalances in the basal ganglia motor pathways. Given its essential role in promoting neuronal survival and meditating synaptic plasticity in the motor system, BDNF might have an important impact on the pathophysiology of neurodegenerative diseases, such as PD. In this review, we focus on the role of BDNF in corticostriatal plasticity in movement disorders, including PD and dystonia. We discuss the mechanisms of how dopaminergic input modulates BDNF/TrkB signaling at corticostriatal synapses and the involvement of these mechanisms in neuronal function and synaptic plasticity. Evidence for alterations of BDNF and TrkB in PD patients and animal models are reviewed, and the potential of BDNF to act as a therapeutic agent is highlighted. Advancing our understanding of these mechanisms could pave the way toward innovative therapeutic strategies aiming at restoring neuroplasticity and enhancing motor function in these diseases.

## 1. Introduction

From early development until adulthood, the vertebrate brain depends on specific sets of signaling molecules for neuronal differentiation, survival, functional maturation, and plasticity. Neurotrophins represent a group of neurotrophic factors involved in these crucial mechanisms. Brain-derived neurotrophic factor (BDNF) is a member of the neurotrophin (NT) family and was the second one of this group of neurotrophic factors to be discovered in 1982 [1,2] after nerve growth factor (NGF), which was identified already in the early 1950’s by Levi-Montalcini [3]. Thereafter, neurotrophin-3 (NT-3), NT-4/5, and NT-6 were discovered as additional members of the NT family [4,5].

Members of the neurotrophin family exert their biological effects by binding to three distinct Trk tyrosine kinase receptors: Tropomyosin receptor kinase (Trk) A, TrkB, and TrkC. NGF binds to TrkA, BDNF and NT-4 activate TrkB, and NT3 can bind to all of them, but it exerts its function mainly through TrkC binding. All these neurotrophins also bind to the p75NTR receptor [6,7,8].

Upon binding of BDNF, full-length TrkB is activated at the cell surface through autophosphorylation and triggers multiple intracellular signaling cascades via protein–protein interactions [9]. The three major pathways activated by TrkB are (1) the phospholipase C (PLC)γ pathway that leads to the production of diacylglycerol and an increase in intracellular calcium, and as a result, activation of Ca^2+^/calmodulin-dependent protein kinase (CAMK) and protein kinase C (PKC); (2) the phosphoinositide 3-kinase (PI3K) pathway that activates AKT, which mediates anti-apoptotic effects; and (3) the mitogen-activated protein kinase/extracellular signal-regulated kinase (MAPK/ERK) pathway that among other cellular effects also activates protein translation [6,10]. By activating these signaling cascades, BDNF plays an important role in the survival of neurons, as evidenced by the prevention of sensory and motoneuron death in cell culture or in vivo after nerve lesions [10,11].

All neurotrophins are expressed in the central and peripheral nervous systems in neuronal and nonneuronal cells. Although they share about 50% amino acid identity with the other members of the neurotrophin family, their expression patterns and levels are distinct [4,12]. Despite having similar expression during embryonic neurogenesis, they show significant temporal and spatial differences, suggesting unique and complementary roles in specific cellular populations and in different functional contexts [13,14].

BDNF mRNA is broadly distributed throughout the central nervous system (CNS) [15,16]. In early embryonic CNS development, BDNF expression levels are very low in mouse and rat brains. These levels then increase during the second postnatal week in rodents in the hippocampus, cortex, hindbrain, and cerebellum [16,17,18]. In adulthood, BDNF expression in the hippocampus and cerebral cortex supports synaptic plasticity and long-term potentiation (LTP) [19,20]. During aging, BDNF and *Ntrk*2 mRNA levels decrease in different brain areas [21].

NGF is expressed in CNS regions such as the hippocampal formation, cortex, olfactory bulb, and basal forebrain. It is essential for the survival and growth of cholinergic neurons [15,22,23]. In the peripheral nervous system (PNS), NGF is critical for the survival, differentiation, and growth of sympathetic and sensory neurons. In adulthood, it is also involved in the response to injury and inflammation [12,24,25,26].

NT-3 is expressed in the developing brain and spinal cord in the CNS, influencing the survival and differentiation of various neuronal populations, including sensory and motor neurons. Although NT-3 expression decreases in adulthood, its expression is still detectable in the hippocampus, cortex, and cerebellum. In the PNS, NT-3 is crucial for the development of proprioceptive neurons and the formation of muscle spindles. In later developmental stages, it also aids in the regeneration of peripheral nerves after injury [27,28,29,30].

The regulation of the expression of neurotrophins at the mRNA level depends not only on developmental mechanisms but also on plasticity and repair-related signaling. For example, Meyer et al. (1992) investigated BDNF and NGF mRNA levels after a lesion of the sciatic nerve in adult rats [31]. They found that BDNF synthesis is significantly enhanced in the injured sciatic nerve, even 10 times higher in comparison to NGF. This highlights distinct regulatory pathways for neurotrophins in response to nerve damage. BDNF protein and mRNA levels are also upregulated after various forms of brain injury, potentially contributing to neuroprotection and recovery processes in the CNS. Several studies have shown that BDNF mRNA levels increase in the hippocampus and cortex after ischemia and seizures in animal models [32,33,34,35].

As part of these mechanisms, it has been shown in the vertebrate brain that the mRNA levels of neurotrophins, in particular those of BDNF, are regulated by neural activity [36,37,38]. Upon neuronal stimulation, membrane depolarization leads to the entry of calcium ions (Ca^2+^) through voltage-gated calcium channels and N-methyl-D-aspartate receptors (NMDARs). This influx of calcium then prompts the activation of transcription factors like calcium-response factor (CaRF) and cyclic AMP response element-binding protein (CREB). These transcription factors subsequently bind to specific regulatory regions of the BDNF gene [5]. Physical exercise boosts the levels of BDNF mRNA and protein in the hippocampus and other brain regions [39]. Elevated BDNF levels have been associated with enhanced LTP, motor learning, and cognitive improvement, as well as protective effects against chronic stress, aging, and neurodegeneration. This has been demonstrated in both animal models and human studies [5,18,39,40,41,42,43]. Mice performing voluntary physical exercise in the running wheel for 3 days showed increased levels of BDNF expression in cortical layers II/III and V and in the striatum in comparison to sedentary mice [18]. Conversely, inhibiting BDNF activity in the hippocampus abolishes the positive effects of exercise on memory performance, and a reduction of 50% in BDNF levels negatively affects motor learning, which further supports the evidence that BDNF plays a significant role in modulating hippocampal and corticostriatal plasticity [18,40,41,44]. The effects of exercise in human studies employing different types of physical training have shown increased levels of BDNF in blood serum that correlate with positive effects in pathological and physiological conditions [45,46,47]. For example, exercise therapy has proven to be effective in raising BDNF levels in the blood and attenuating motor symptoms in patients with Parkinson’s disease [48]. Overall, neural activity triggered by physical exercise appears to play dual roles in which the neuronal activity regulates BDNF expression, and BDNF modifies and regulates synaptic activity, inducing neuroprotective effects.

BDNF can modulate synaptic plasticity by specifically enabling synapses to undergo activity-induced LTP or long-term depression (LTD) [38,49,50,51]. In neonatal rat hippocampal slice cultures, the addition of recombinant BDNF enhanced the induction of LTP at CA3-CA1 synapses by increasing the efficiency of presynaptic neurotransmitter release during high-frequency stimulation (HFS) [52]. The facilitation of LTP by BDNF is also directly associated with structural plastic changes, such as spine enlargement and the extension of dendritic arbors [53,54,55], and it promotes hippocampal neurogenesis in adult rat brains [56].

The functional role of BDNF in LTP was further validated using BDNF knockout mouse models, where both homozygous and heterozygous mice exhibited significantly reduced LTP in the hippocampus [57,58]. Korte et al. (1995) showed that exogenous BDNF application mimics activity-dependent synaptic modifications [57]. This study also demonstrated that a partial loss (~50%) of BDNF is sufficient to impair LTP expression in the hippocampus.

These findings go along with observations of cognitive and behavioral alterations in mice and patients in which BDNF expression levels are reduced by deletion or mutations in one allele of the BDNF genes or by deletion of its receptor TrkB. The severity of cognitive alterations in BDNF^+/−^ mice seems to vary between different studies [59,60,61,62]. However, recent analyses indicate that both learning-facilitated LTD and spatial reference memory are impaired in BDNF^+/−^ mice, and conversely, a positive correlation between hippocampal BDNF protein levels and learning performance in long-term memory tests was detected [63,64]. This indicates that BDNF is critical for regulating neural plasticity in the hippocampus. Yet, these observations still raise the question of whether synaptic plasticity mechanisms in brain regions other than the hippocampus are equally responsive to changes in BDNF levels. This question is particularly significant when considering BDNF’s functional role in motor control, specifically concerning BDNF that is derived from corticostriatal and dopaminergic afferents to striatal neurons.

## 2. Effects of TrkB and BDNF Knockout on the Motor System

The BDNF/TrkB signaling pathway has been extensively investigated, and studies from knockout (KO) models show that its disruption induces severe neurological symptoms [65,66] that also affect the striatum. The findings from these models are discussed below.

### 2.1. TrkB Knockout

TrkB is expressed from multiple transcripts of the neurotrophic receptor tyrosine kinase 2 (*Ntrk*2) gene, leading to two major isoforms of the TrkB receptor: Full-length TrkB and truncated TrkB, which lacks the catalytic kinase domain [67,68]. The first evidence for the indispensability of TrkB signaling in the CNS came from a mouse model where a replacing cassette was introduced into a region encoding the catalytic domain of TrkB. This resulted in the inactivation of full-length *Ntrk*2 mRNA and protein, whereas expression of the truncated isoform was only slightly reduced. Mice carrying this mutation on both alleles survive until birth, but most animals die by P1 due to their inability to feed. Furthermore, homozygous mice show abnormalities both in the central and the peripheral nervous system. Although heterozygous mice show reduced full-length TrkB levels, they did not exhibit neuronal deficiencies in the structures investigated [69].

### 2.2. BDNF Full Knockout

BDNF KO mice display similar impairments as mice with TrkB depletion, although they appear to have a milder phenotype [70,71]. Targeted disruption of the BDNF gene resulted in the depletion of BDNF mRNA in homozygous mice and about half the levels could be detected in heterozygous compared to wildtype mice [70]. In this model, BDNF^−/−^ animals showed a reduced size compared to their wildtype littermates, but most of them survived until postnatal week two, in contrast to the lethality at P1 for *Ntrk*2^−/−^ mice [69]. BDNF^−/−^ mice show coordination, balance, and movement deficits, and the numbers of neurons were significantly reduced in sensory and parasympathetic ganglia, such as the trigeminal, vestibular, and nodose ganglion. No differences were seen in the number of dopaminergic neurons in the midbrain of BDNF^−/−^ mice. Interestingly, no deficiencies could be observed in facial motor nuclei, whereas there was a marked loss in this cell population in *Ntrk*2^−/−^ mice [69].

LTP was markedly reduced in Schaffer collaterals of the BDNF KO model when the CA1 region was stimulated in hippocampal slices. This deficit could be rescued by the delivery of endogenous recombinant BDNF to the slices for 5–8 h [57,72]. Interestingly, the reduction of BDNF levels in hippocampal slices of BDNF^+/−^ mice was sufficient to significantly impair hippocampal LTP formation. After tetanic stimulation of hippocampal slices from BDNF^+/−^ mice, LTP responses appear indistinguishable from those from BDNF^−/−^ mice [57,72].

In another model, mice homozygous for BDNF KO also show movement defects [71]. BDNF^+/−^ animals do not show gross behavioral abnormalities and have a survival comparable to wildtype animals. Similar to the model introduced by Ernfors et al. (1994), the mice generated by Jones et al. (1994) showed no alterations in motoneuron numbers in the spinal cord, but sensory neuron number was significantly reduced in the dorsal root spinal ganglia. Furthermore, the number of calbindin-positive neurons was massively reduced in the striatum of BDNF^−/−^ animals. Calbindin is thought to be expressed mainly in striatal spiny projection neurons (SPNs) [73], but these neurons seem to develop in comparable numbers in BDNF^−/−^ and BDNF^+/+^ mice, as GABA-staining revealed no differences in cell numbers in the striatum [71]. This finding gave concrete indications about differences in Ca^2+^ metabolism and thus altered neuronal activity in the striatum of BDNF^−/−^ animals, possibly contributing to the severe motor phenotype observed.

Another approach to investigate the roles of BDNF in the brain, which has been widely used, is the conditional KO in a cell type-specific manner.

### 2.3. BDNF Conditional Knockout (Wnt1–Cre)

Proteins of the WNT family are small signaling molecules that are implicated in cell proliferation, migration, and carcinogenesis and, furthermore, possess regulatory functions in mouse embryonal development [74]. It has been shown that Wnt1 expression during embryonic stages is mostly confined to regions of the midbrain and hindbrain (MHB) [74,75]. Consequently, a mouse model with Cre expression from the Wnt1 promotor was generated to alter gene expression in an MHB-specific manner [75].

Crosses of these mice with BDNF^lox/+^ mice lead to animals lacking BDNF in the MHB region [76]. As early as embryonic day 16.5, low levels of BDNF expression can be observed in the substantia nigra pars compacta (SNc), which partially overlaps with tyrosine hydroxylase (TH) staining, showing that a subset of dopaminergic neurons in the SNc expresses BDNF. In the SNc, 80–90% of TH-positive neurons are positive for Wnt1–Cre expression, meaning that BDNF can be effectively depleted in dopaminergic neurons of the SNc. These mutant mice do not show motor abnormalities when observed in their home cage, but a decrease of performance in the accelerating rotarod test at 4 weeks of age was apparent, coinciding with the reduction of BDNF in the MHB of both heterozygous and homozygous BDNF KO animals at young postnatal stages (P7–P9). Also, the animals show a hindlimb clutching phenotype at 1 month, as assessed by the tail suspension test, which is more pronounced at 4 months of age. Furthermore, the number of TH-positive cells was reduced in the SNc of homozygous animals at postnatal stages (P0, P21, and P120), which is also mirrored by reduced TH levels in the striatum at P35. Interestingly, although BDNF is already greatly reduced in heterozygous animals, the motor performance appears normal, and deficits become only apparent after homozygous BDNF KO in the MHB. Similar observations are made for the number of TH-positive neurons, where heterozygous and wildtype animals are indistinguishable at every age investigated, meaning that the residual BDNF is sufficient to prevent the loss of TH expression in the SNc. Conclusively, these data show that BDNF is required for functional maintenance of nigrostriatal dopaminergic neurons during postnatal development [76].

### 2.4. BDNF Conditional Knockout (Emx1–Cre)

EMX1 is a homeodomain protein that is expressed mainly in cortical excitatory neurons and glia cells [77]. Emx1^IREScre^ mice were intercrossed with BDNF^lox/+^ mice to examine the role of BDNF KO specifically in cortical neurons [78]. This model shows expression of Cre recombinase as early as embryonic day 10.5. Thus, BDNF is expected to be depleted throughout the late embryonic and the whole postnatal period. The survival of these animals appears normal up to mid- to late adulthood, but there might be a general shortening of lifespan at ages from 12 months onwards. As a consequence of BDNF depletion, cortical thickness and pyramidal neuron morphology of cortical layer II/III was altered during development at 2 weeks and 5 weeks of age [78]. Also, the volume of the hippocampus was not altered, but both cortical and striatal volume was significantly reduced in Emx-BDNF^KO^ animals compared to wild-type controls. Moreover, SPN area and spine density were markedly reduced in this model [79].

General locomotor activity in these mice was reduced as assessed by the open field test, but anxiety was not altered (open field and elevated plus maze test) [80]. The tail suspension test revealed a clasping phenotype at 1 month of age, which was even more pronounced at 4 months of age. Also, using the accelerating rotarod test, no differences in motor performance were observed at 1 or 2 months of age, but at 4 months of age, Emx-BDNF^KO^ animals showed a slightly decreased latency to fall compared to wildtype controls [79]. Emx-BDNF^KO^ mice furthermore showed a reduced capability of learning several cognitive tasks. Impairments were observed in spatial learning (Morris water maze) and discrimination learning (T-maze), whereas anxiety and fear conditioning were not altered [80].

### 2.5. BDNF Conditional Knockout (Tau–Cre)

The TAU protein is expressed in postmitotic neurons [81], and therefore, Cre expression under the control of the Tau promotor makes it possible to introduce perturbations to all CNS neurons after critical stages of development when neurons are generated and migrate. Introducing a BDNF knockout specifically in Tau-expressing neurons led to an almost complete depletion of BDNF protein in the brain of 2-month-old mice, whereas the survival of these mice appeared normal compared to wild-type controls [16]. Total brain volume in the Tau–BDNF^KO^ animals was reduced, with no changes in the hippocampus but significant reductions in the striatum. Furthermore, dendrite length, dendrite number as well as spine density in SPNs was massively reduced in these animals. In contrast, hippocampal morphology was mostly normal, with slight alterations in the types of dendritic spines expressed in CA1 hippocampal neurons. At 8 weeks of age, the Tau–BDNF^KO^ animals showed a clasping phenotype in the tail suspension test, and exploratory behavior was markedly reduced (dark/light exploration test) [16].

In summary, both the Emx–BDNF^KO^ and the Tau–BDNF^KO^ show massive degeneration of striatal neurons with less degeneration in hippocampal structures, indicating that striatal neurons are more sensitive to BDNF depletion than hippocampal neurons, and there are less efficient mechanisms in the striatum to compensate for postnatal BDNF loss than in the hippocampus.

## 3. Anterograde Transport and Release of BDNF from Corticostriatal Projection Neurons

The striatum is a subcortical structure of the basal ganglia, which receives mainly glutamatergic inputs from the cortex and dopaminergic inputs from the substantia nigra (SN) [82,83]. The corticostriatal synapses are located on the heads of dendritic spines of SPNs. These neurons comprise around 90% of the total population of neurons in the striatum and project to the SN and globus pallidus. The neuronal activity of striatal neurons is primarily controlled by excitatory inputs from the cortex [84,85].

Several studies have shown that BDNF is essential for the survival and functional differentiation of striatal neurons in vitro and in vivo [86,87,88]. It also plays a crucial role in synaptic plasticity at corticostriatal synapses, eliciting LTP and LTD and modulating functions such as learning, adaptation, and motor coordination [38,89,90]. Despite its important functions in the striatum, multiple studies have demonstrated that BDNF is not directly expressed in this brain area. Interestingly, BDNF protein levels are high in the striatum, but BDNF mRNA is virtually absent [91]. BDNF is synthesized in other brain regions, such as the cortex, SN, amygdala, and thalamus, and it is transported anterogradely along axonal processes and stored within presynaptic terminals of excitatory neurons [5,92,93,94]. Previous studies have used antibodies reacting either with BDNF or pro-BDNF to study the subcellular distribution of BDNF. The BDNF protein is localized in large, dense core vesicles in presynaptic terminals of excitatory neurons. This confirms an anterograde mode of action of BDNF [93,94].

To make BDNF available in the striatum, the activation of presynaptic NMDA receptors and the subsequent prolonged elevation of Ca^2+^ are required. This has been demonstrated in mouse neurons, where genetic depletion of either BDNF or NMDA receptors resulted in the abolition of LTP at corticostriatal synapses. Specifically, the elimination of BDNF expression through Cre–loxP deletion of the BDNF gene in M1 cortical axons caused reduced basal synaptic transmission and lower excitatory postsynaptic potential (EPSP) responses during theta burst stimulation. Additionally, the deletion of the GluN1 subunit from NMDA receptors in cortical neurons using a conditional gene strategy impaired LTP at corticostriatal synapses [89].

**Figure 1:**
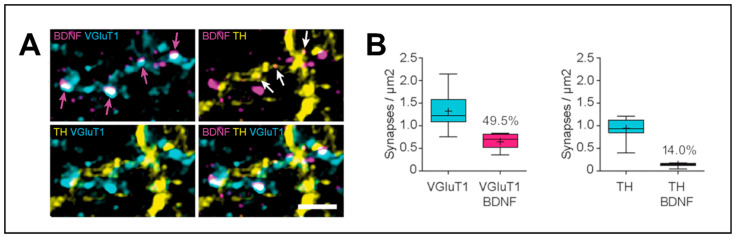
Motor cortex and substantia nigra contribute differently to striatal presynaptic brain-derived neurotrophic factor (BDNF). (**A**) High-resolution light microscopy of BDNF-containing terminals reveals high overlap with Vesicular glutamate transporter 1 (VGluT1) staining (magenta arrows), whereas overlap with tyrosine hydroxylase (TH)-immunoreactive structures was less frequently observed (white arrows). Scale bar: 1.5 µm. (**B**) Quantification of VGluT1-positive synapses (left, blue) and VGluT1-BDNF double-positive synapses (left, magenta). Also the numbers of TH-positive synapses were quantified (right, blue), as well as TH-BDNF double-positive synapses (right, magenta). Figure modified from Andreska et al. (2020) [18] (licensed under CC BY 4.0).

BDNF protein found in the striatum is originally synthesized and anterogradely transported from the cell bodies of cortical neurons but also from neurons of the SNc, amygdala, and thalamus (Figure 1) [79,92]. Both the conditional ablation of BDNF expression in SN and cortex lead to developmental deficits in striatal neurons and motor dysfunction [95]. The Wnt1–BDNF KO mice showed that the lack of BDNF from the midbrain-hindbrain causes poor motor performance in mice that correlates with deficits in dopaminergic neurons [76]. Similarly, conditional BDNF KO in which BDNF is depleted in all neurons led to a significant reduction in striatal volume, together with dendritic loss in striatal SPNs [16]. This indicates that BDNF from cortical glutamatergic and possibly also from dopaminergic afferents from the SN is necessary for the postnatal dendritic growth and maintenance in striatal neurons [16].

**Figure 2:**
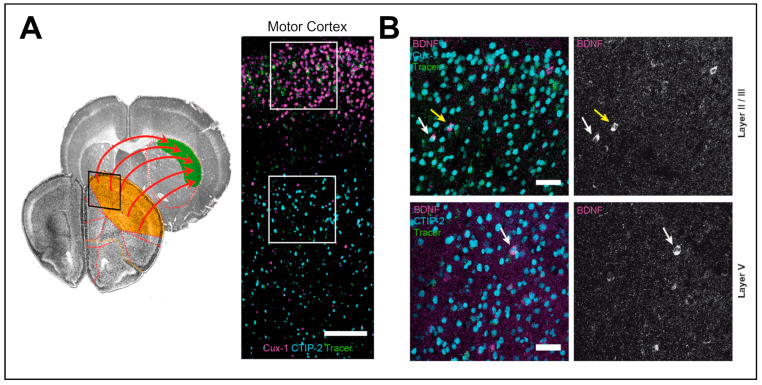
Dorsal striatal BDNF is delivered from Layer II/III and V of the motor cortex. (**A**) BDNF-expressing striatal afferents have their origin in the motor cortex (left). Retrograde tracing from the dorsal striatum identifies neurons in Layer II/III (Cux1-positive) and Layer V (Ctip-2-positive) of the motor cortex (right). Scale bar: 150 µm. (**B**) BDNF-expressing neurons in the motor cortex are also positive for the retrograde tracer injected into the dorsal striatum (white arrows). Not all BDNF-positive neurons were positive for the retrograde tracer (yellow arrow). Scale bar: 50 µm. Figure modified from Andreska et al. (2020) [18] (licensed under CC BY 4.0).

The anterograde axonal transport and release of BDNF from axonal corticostriatal terminals appear particularly important for neuroplasticity in projection neurons in the striatum. Retrograde tracing from the dorsolateral striatum showed that layer II/III and V neurons in the motor cortex express BDNF, which plays a crucial role in promoting postsynaptic changes necessary for motor learning and LTP induction at corticostriatal synapses (Figure 2) [18,96]. The levels of BDNF expression in the cortex are age-dependent, being higher at earlier developmental stages where motor skills are learned and acquired. Likewise, BDNF levels appear reduced during aging when the demand for motor learning is lower [18].

These findings furthermore support that the survival of striatal neurons depends on cortical BDNF. This important role of BDNF in the striatum raises the question about its role in several neurological diseases involving the basal ganglia and what molecular and cellular mechanisms contribute to motor dysfunction.

## 4. The Role of BDNF in Corticostriatal Projection Neurons for Motor Learning

### 4.1. BDNF Is Necessary for LTP at Corticostriatal Synapses

Due to the striking effect of motor activity on BDNF upregulation and the role of BDNF in mediating synaptic plasticity, the importance of BDNF for motor learning has received more attention recently. For the learning and execution of new motor tasks, the motor cortex and the dorsolateral striatum play a central role [18,97,98,99,100]. As mentioned above, the majority of BDNF from cortical afferents is delivered to the dorsolateral striatum via corticostriatal projections from the motor cortex, providing another link between these two structures for motor tasks [18,92]. BDNF acts to mediate LTP in the dorsal striatum. Here, HFS in the cortex of ex vivo brain slices induces rapid LTP in control SPNs but not in SPNs of slices treated with a BDNF scavenger protein. When adding exogenous recombinant BDNF to the brain slices, isolated postsynaptic NMDAR responses to cortical HFS are increased in striatal SPNs [96]. The essential role of BDNF in corticostriatal LTP was confirmed by another study showing that LTP induction by cortical theta-burst stimulation in the cortex of ex vivo brain slices was also blocked by a BDNF scavenger protein. Depleting BDNF specifically in M1 cortical axons through an adeno-associated virus (AAV) -mediated approach, theta-burst stimulation-induced LTP at corticostriatal synapses was also absent. Interestingly, BDNF secretion at corticostriatal synapses is dependent on axonal NMDARs of the presynaptic neuron, and neuronal activity is needed to efficiently release BDNF from presynaptic terminals [89].

### 4.2. Motor Activity Regulates BDNF Levels in the Brain

BDNF can be detected in many regions of the CNS [2,15,37,101,102,103,104]. Generally, BDNF mRNA levels are upregulated upon neuronal activity, which is a prerequisite for the increase of de novo BDNF synthesis [37,102,104,105]. This regulation is mediated by the transcription factor CREB, whose phosphorylation and resultant activation are increased by Ca^2+^ influx into the cytosol of neurons, indicative of neuronal activity [106].

Examples of behavioral mechanisms for the upregulation of BDNF in rodents are environmental enrichment and physical exercise. In experiments with adult rats, enrichment of their home cages significantly increases BDNF mRNA levels in pyramidal cells of the hippocampal CA1 region, which coincides with improved spatial memory performance [103]. Another prominent example of activity-dependent regulation of BDNF is the upregulation of BDNF levels upon motor activity. However, regulation of BDNF expression in the motor cortex after exercise has received less attention when compared with the hippocampus. After 3 days of voluntary exercise, BDNF was upregulated in mice both in the striatum as well as in layers II/III and, to a lesser extent, in layer V of the motor cortex. Tracing experiments showed that this BDNF increase also occurs in cortical neurons projecting directly to the dorsal striatum [18].

As evidence for the activity-related regulation of BDNF in the motor system is limited, looking at other brain regions can provide helpful insights. However, it is not clear whether the mechanisms observed in the hippocampus also apply to the situation in the motor cortex.

After 2–7 days of voluntary physical exercise, rats show increased BDNF mRNA in the CA1 and CA3 regions of the hippocampus but also in Layer II/III and V/VI of caudal areas of the neocortex [39,107]. This is also reflected by increased BDNF protein levels [108]. Furthermore, BDNF mRNA levels also correlate with the amount of physical activity [39,107]. In these studies, short (2 days) physical exercise led to an increase of BDNF mRNA in the frontal cortex, but with longer exercise up to 7 days, BDNF levels decreased again to the basal levels found in sedentary rats [39].

Since the BDNF gene possesses different promotors [109], the differential usage of these promotors can drive tissue- and stimulus-specific expression of BDNF [104,105]. In the hippocampus, total BDNF mRNA is upregulated as early as 6 h after physical exercise. Exon I mRNA is upregulated at 6 h and enhanced at 12 h, and Exon II mRNA upregulation is only observed after 12 h. Exon III and IV are not enhanced even after 12 h [110]. This also shows a time-dependent usage of different BDNF promotors following physical activity. Another study found BDNF upregulation in rat hippocampus only in Exon I after 1 day of voluntary exercise and in Exon I, II, and V after 28 days. BDNF protein was only increased after prolonged exercise of 14–28 days. However, this study did not include pre-training of the animals for their exercise paradigm and, therefore, cannot be directly compared to other studies [111]. In mice, hippocampal BDNF protein increases after 7 days of exercise and is maintained with prolonged exercise. Exon II mRNA in the hippocampus increases with exercise in young animals (2 months) but not in older animals (15 or 24 months). Other promotor-specific mRNA species were not upregulated in this study [112]. Hippocampal BDNF upregulation is sustained for several days after the exercise, and similar BDNF levels were observed when comparing daily training with training on alternating days in rats [113]. Although it is generally accepted that motor activity leads to an increase of brain BDNF levels, the exact time course and the impact of the type and amount of the exercise, and the response on BDNF expression in different brain areas is less clear. For example, conditions of motor training that elevate the number of BDNF-positive neurons in layer II/III and layer V of the motor cortex in 12 weeks old mice do not have an effect on BDNF expression in neurons of layer VI of the somatosensory cortex [18].

### 4.3. Implications of Cortical BDNF for Motor Learning

Plastic synaptic alterations are a prerequisite for learning, and the involvement of BDNF in corticostriatal LTP and activity-dependent BDNF upregulation in the motor system makes it likely that BDNF-mediated corticostriatal LTP is directly involved in motor learning.

Exercise training prior to learning a new motor task leads to improved learning compared to non-exercising animals [114], and given that BDNF expression is upregulated after motor activity, this effect is potentially attributable to an increase of cortical and striatal BDNF. Evidence for the direct involvement of BDNF in motor learning comes from a study using conditional KO of BDNF using Cre-mediated recombination under the neurofilament light chain (NF-L) promoter for one allele of the BDNF gene, which leads to ~50% reduction of BDNF in pyramidal neurons of the cerebral cortex. After this conditional BDNF depletion, 3-month-old mice showed severe impairment in the learning of a new motor task, as measured by their performance in the irregular ladder-rung walking test [18].

Taken together, this shows that BDNF is a crucial mediator for motor activity and motor learning and indicates that a lack of BDNF potentially impairs corticostriatal synaptic plasticity needed for these learning processes and the correct functioning of the motor system.

## 5. Alterations of BDNF in Parkinson’s Disease

The most obvious symptoms observed in patients with Parkinson’s Disease (PD) are motor impairments, and it is clear that the progressive degeneration of nigrostriatal dopaminergic projections underlies many of the symptoms observed in the disease. Although the pathological dopamine (DA) depletion and the resulting imbalance of neural activity in the striatal direct and indirect pathways are implicated in the motor symptoms in PD, the mechanisms of how DA depletion in the striatum causes these symptoms are still not fully understood [115].

Given the important role of BDNF for motor learning and motor control, it is tempting to speculate that disturbed BDNF/TrkB signaling and, thus, dysregulated synaptic plasticity contribute to PD pathology.

### 5.1. Alterations of BDNF mRNA and Protein Expression

On the BDNF mRNA level, in situ hybridization revealed a significant reduction of BDNF mRNA in the SNc of PD patients, and this reduction was not due to mere cell loss, as the remaining neurons in the SNc also expressed significantly less BDNF compared to healthy controls [116]. Animal models of PD showed similar results. After 6-Hydroxydopamine (6-OHDA) injection into the ventral mesencephalon or the ascending medial forebrain bundle (MFB), rats showed substantially reduced BDNF mRNA in the SNc and ventral tegmental area (VTA) as visualized by in situ hybridization [117]. Similar observations were made after the injection of 6-OHDA directly in the SN [118]. Although of great importance for understanding the role of BDNF in the motor system during PD development, these studies did not investigate BDNF mRNA levels in the motor cortex of these PD rodent models.

The first evidence for dysregulated BDNF protein related to PD comes from measurements of BDNF expression in patients with PD. Immunohistochemical staining for BDNF in the SNc of human postmortem tissue revealed a massive loss of BDNF expression in PD patients [119]. This finding was also confirmed by ELISA measurements of BDNF levels in the SN of patients with PD [120].

The same study also reported reduced BDNF protein in the striatum of PD patients [120]. In the 1-methyl-4-phenyl-1,2,3,6-tetrahydropyridine (MPTP) monkey model, BDNF protein levels were reduced in the striatum of the lesioned side [121]. There also appears to be a slight decrease of BDNF protein in the frontal cortex of PD patients, although the differences to the healthy controls were not statistically significant [120]. Taken together, these few studies indicate that BDNF levels appear relatively normal in the motor cortex during the development of PD but decline in the striatum. One possibility is the disturbed anterograde transport of BDNF from corticostriatal afferents.

### 5.2. Altered Anterograde Transport of BDNF in Corticostriatal and Dopaminergic Projections

Defects in axonal transport have been implicated as another mechanism for the contribution to PD pathology. Most of this evidence is linked to α-Synuclein, as this protein is involved in endocytic pathways and axonal transport. More specifically, expression of PD-associated mutated α-Synuclein forms in cultured cortical neurons leads to defective axonal transport of α-Synuclein itself [122]. In the same study, axonal mitochondria transport was not impaired upon mutations of α-Synuclein [122], indicating that the axonal transport defect might be specific for some cargoes and does not reflect a general defect of axonal transport. Regarding dopaminergic neurons, the expression of mutant (A53T) human α-synuclein in the SN of rats leads to a massive dysregulation of multiple axonal transport proteins in the striatum [123]. Furthermore, kinesin and dynein levels were decreased in SN neurons of patients with PD in the dopaminergic projections, with a stronger reduction observed in neurons with α-Synuclein inclusions [124]. Overexpression of mutant (A30P) human α-synuclein in the SN of a rat model leads to similar reductions of kinesin and dynein in dopaminergic neurons [124]. Evidence of how α-synuclein-associated impairments of axonal transport could contribute to PD development is summarized in the review by Volpicelli-Daley, 2017 [125].

As mentioned above, most of the striatal BDNF is anterogradely transported from the motor cortex via corticostriatal glutamatergic afferents (Figure 1 and Figure 2) [18,92]. One study reported a link between α-Synuclein and defective BDNF trafficking. Here, cortical embryonic (E18) neurons transfected with human wild-type α-synuclein showed impaired retrograde axonal transport of BDNF after exogenous application to the axon, together with an activity increase of the endocytic proteins Rab5 and Rab7 [126]. Although there appear to be transport defects, anterograde endogenous BDNF transport was not examined in this study. Another recent study suggests that upon α-Synuclein accumulation in the α-Synuclein preformed fibril (PFF) rat model, BDNF anterograde transport is disturbed both in nigrostriatal and corticostriatal projections, despite unaltered total BDNF levels in these structures. In this model, deep brain stimulation (DBS) of the subthalamic nucleus (STN) partially rescued the proposed BDNF transport impairments [127].

### 5.3. Release of BDNF in the Striatum

Apart from a potential deficit in BDNF axonal transport, another possibility for the contribution to PD development could be an altered BDNF release at presynaptic sites. Various stimuli can lead to BDNF release, most of them associated with increased neuronal activity and neuron depolarization in a Ca^2+^-dependent manner (reviewed by Brigadski and Leßman, 2020 [128]). More specifically, it was found that in rat primary nodose-petrosal ganglion cell cultures, HFS at 50 Hz leads to a massive release of BDNF from these cells, whereas stimulations at lower frequencies were less effective in triggering BDNF release [129]. Similarly, in rat primary hippocampal cultures, electrical field stimulation leads to rapid BDNF release, whereas again, higher frequencies (100 Hz and theta-burst stimulation) were more effective. The effects of these stimulations were dependent on extracellular Ca^2+^ and voltage-gated sodium channels, meaning action potentials were needed for BDNF release from presynaptic terminals [130]. Interestingly, theta-burst stimulation is also effective in inducing hippocampal LTP and triggers neuronal activity and classical action potential firing [131,132,133]. Until today, there is no direct evidence for an impaired neuronal BDNF release in PD, but beneficial effects of exogenously applied BDNF on PD pathology hint at reduced BDNF/TrkB signaling in the pathophysiology of PD, which possibly could involve defective synaptic BDNF release.

### 5.4. Potential Effects of Deep Brain Stimulation and Neuronal Activity Modulation

Besides the most common treatment of PD, L-DOPA administration, DBS is widely used for the treatment of the motor symptoms of the disease. Usually, the sites of implantation for DBS electrodes in PD patient brains are the STN and the internal Globus Pallidus (GPi) [134]. DBS in PD is then achieved by applying HFS (>100 Hz) electrical stimulation to the implanted areas [135]. The neuronal activity pattern generated by DBS is generally complex, exhibiting both activating and inhibitory components [136]. Although the exact mechanisms of how DBS ameliorates PD symptoms are still not known, there are several hypotheses on the function of DBS. One concept is, that DBS enhances neuronal activity, as in vivo recordings indicate, that the output of either the GPi or the STN is increased upon DBS in these structures [137,138]. Although not directly projecting to the Striatum or SN, GPi or STN DBS could affect these structures by modulating the activity of neuronal networks, specifically the basal ganglia network in this case. As an increased neuronal activity, in particular with a frequency of 100 Hz, is also linked to enhanced BDNF release (see above), stimulation of BDNF-expressing neurons with high frequencies can lead to synaptic BDNF release, either directly at the output of the DBS electrode-implanted structures or also at distant sites that receive input from neurons that are electrically stimulated from the DBS electrodes. For instance, DBS could increase BDNF release in the striatum via different pathways, either at corticostriatal synapses of the direct or indirect pathway or also at synapses inside the STN via the hyper-direct pathway projecting directly from the cortex to the STN. There is limited evidence that DBS indeed enhances BDNF release. It has been shown that BDNF protein levels are higher in the striatum, the SN, and the motor cortex after DBS of the STN. However, this increase is possibly attributable to increased production of BDNF, as BDNF mRNA was also increased in the SN of stimulated animals [139]. Also, STN-DBS partially rescues BDNF transport deficits in nigrostriatal and corticostriatal projections in the α-Synuclein PFF model [127]. Taken together, an enhanced production, transport, and possibly secretion of BDNF could add to the beneficial effects of DBS [140].

### 5.5. BDNF as a Therapeutic Agent for Parkinson’s Disease

As described above, BDNF levels are lower in several structures of the motor system in PD patients and in vivo disease models. Additionally, BDNF transport and release may be impaired. Thus, targeting BDNF/TrkB signaling could be a therapeutic approach for the treatment of PD symptoms.

Studies have been performed examining the potential therapeutic benefits of BDNF in PD. The neurotrophic properties of BDNF are well characterized and most of the studies using BDNF as a therapeutic agent indicate a neuroprotective effect of BDNF as the mechanism of action. BDNF was also found to act as a neurotrophic factor for SN dopaminergic neurons [141,142], and it promotes DA uptake in cultured midbrain cells [142,143]. Depletion of BDNF strongly affects striatal neurons that play a central role in the motor control circuit. Adding BDNF to cells in vitro reduces 1-methyl-4-phenylpyridinium (MPP^+^) and 6-OHDA toxicity in SH-SY5Y cells and primary dopaminergic SN neurons [141,144]. In the MPTP monkey model of PD, intrathecal delivery of human recombinant BDNF before and in parallel to MPTP injection attenuates PD symptom progression, which correlates with reduced SN dopaminergic cell degeneration compared to animals without exogenously applied BDNF [145]. Another study in the 6-OHDA rat model found that BDNF gene delivery and resulting BDNF expression in the SN leads to a reduction in dopaminergic neuron degeneration, partially rescues DA levels in the striatum, and restores locomotor defects [146]. Furthermore, systemic administration via intraperitoneal injection of the TrkB agonist deoxygedunin protects nigrostriatal dopaminergic neurons from MPTP and 6-OHDA toxicity in mice and rats [147]. Oral administration of the TrkB agonist CF3CN rescues SN dopaminergic neuron number in an MPTP mouse model through the inhibition of δ-secretase and the resulting decrease of α-Synuclein cleavage and fragmentation [148].

However, it should be clearly differentiated between the neurotrophic or neuroprotective effects of BDNF in the prevention of SN neuron degeneration and the direct action of exogenously introduced BDNF on neuron signaling and plasticity induction. As striatal SPNs generally show no degeneration during PD, the action of BDNF is likely to be distinct from the neurotrophic effects seen in nigral dopaminergic neurons. In the unilateral 6-OHDA rat PD model, artificial BDNF expression via direct gene transfer into striatal neurons rescues abnormal rotational behavior observed in these animals and partially protects against 6-OHDA-mediated nigrostriatal neuron degeneration [149]. Furthermore, administration of BDNF to rat striatum in vivo enhances neuropeptide expression both in the intact and the DA-depleted striatum [150].

Although elevating BDNF activity seems like a potential strategy for PD therapy, direct delivery of the BDNF protein appears problematic due to the difficulty of efficiently delivering it to the brain [151]. Feasible methods of BDNF delivery to the brain would be systemic administration or intrathecal injection, but here, effects can occur in a brain-wide, non-specific manner. Due to the doses needed to elicit effects in the striatum, potential side effects and toxicity cannot be ruled out [151,152]. Another possibility for boosting BDNF levels in the brain is gene therapy, where viral vectors are used to induce the expression of a certain protein in a region- or cell-type-specific manner [153]. Although this approach appears as a promising possibility for employing BDNF as a therapeutic strategy, no clinical trials in human PD patients have been conducted so far.

As mentioned above, another way of enhancing BDNF levels in the brain is physical activity, and it has indeed been shown that exercise possibly slows PD progression and reduces PD-related symptoms [154,155]. Physical exercise modulates neuronal circuits in multiple ways, and one possibility of how physical exercise could ameliorate PD symptoms is via modulation of reactive oxygen species (ROS) metabolism and ferroptosis [156]. Ferroptosis is a form of regulated cell death, which is iron-dependent and has been suggested to play a role in the nigrostriatal neurodegeneration observed in PD [157]. A key regulator of ferroptosis, nuclear factor erythroid 2-related factor 2 (NRF2), was found to be upregulated in rat striatum after physical exercise, and it has been reported that this protects against MPP^+^-induced neurodegeneration of dopaminergic neurons and ameliorates motor manifestations in a rotenone rat model of PD [158,159,160]. Furthermore, it has been proposed that BDNF signaling increases NRF2 levels in astrocytes via p75NTR [161,162]. However, the direct link between BDNF upregulation after exercise and modulation of ferroptosis is still not fully understood. Furthermore, BDNF blood serum levels increase after physical exercise [48,163,164]. Given the crucial role of BDNF for intact motor function [18] and the observations that BDNF levels are rapidly increased upon motor exercise (see above), it is likely that exercise-induced BDNF upregulation in corticostriatal projections contributes to the beneficial effects of exercise on PD symptoms. However, direct evidence for this is missing, and further research is required to define the precise role of BDNF in this context.

## 6. Alterations of TrkB Signaling in Parkinson’s Disease

### 6.1. Alterations of Ntrk2 mRNA and TrkB Protein Expression

Besides BDNF itself, its receptor TrkB has also been implicated in PD disease development. In human postmortem tissue, NTRK2 mRNA expression was not altered in the remaining SNc neurons of PD patients [165], whereas, in the 6-OHDA rat model, the number of TrkB-positive dopaminergic neurons in the SNc was reduced in an early presymptomatic stage before neuron degeneration occurs [166]. In another study, *Ntrk*2 mRNA was not changed in the SN after MFB transection in rats up to 14 days after the lesion [167].

In the striatum, *Ntrk*2 mRNA levels increase in a rat model ipsilateral to 6-OHDA injection into the MFB or MFB transection [167,168,169]. After MFB 6-OHDA injection or MFB transection in rats, also TrkB protein is increased in the striatum ipsilateral to the lesion [170,171]. In a 6-OHDA MFB mouse model, chronic treatment with L-DOPA or a dopamine D1 receptor (DRD1) agonist but not with a DRD2 agonist led to increased total TrkB levels in the striatum of the lesioned side [169].

Interestingly, whereas BDNF levels generally are reduced in both SNc and striatum of PD patients and disease models (see above), TrkB expression appears to be decreased or unchanged in the SNc but increased in the striatum.

### 6.2. TrkB Depletion Induces Cell Loss in the Substantia Nigra

Interestingly, heterozygous depletion of TrkB results in the loss of SNc neurons in aged rats (21–23 months), which is not observed at a younger age (6–8 months) [172]. In mice, TrkB depletion leads to a progressive loss of dopaminergic SNc neurons, beginning at 12 months of age. Concomitantly, dopaminergic terminal size and number are reduced in the striatum of these animals at 18 months of age [173]. The late effects of TrkB depletion on SNc integrity in these models indicate a mechanism that is rather chronic than acute, and it appears that the downregulation of TrkB expression increases the vulnerability of nigrostriatal dopaminergic neurons. This is supported by the finding that in younger mice (8–10 weeks) receiving MPTP treatment, TH-positive cell counts were more drastically reduced after TrkB depletion compared to controls with wildtype TrkB levels [173].

### 6.3. TrkB Interaction with α-Synuclein Is Linked to Pathology in Parkinson’s Disease

Alpha-Synuclein is a protein closely linked to PD pathophysiology, and it was shown that TrkB directly binds to α-Synuclein via its kinase domain, which blocks BDNF/TrkB signaling pathways. In mice, this impaired TrkB activity appears to be at least partially responsible for the nigral dopaminergic cell death observed after α-Synuclein overexpression, which provides another link between PD and BDNF/TrkB signaling [174]. How exactly the association of TrkB with α-Synuclein leads to these observations is not clear, but possibly TrkB sequestration by α-Synuclein, especially α-Synuclein aggregates, could lead to the redistribution of TrkB within the cell and consequently reduce its availability on the cell surface for the binding of BDNF. Generally, a dysregulated subcellular distribution of TrkB in PD seems putative.

### 6.4. Changes in Subcellular Distribution of TrkB and Implications for Corticostriatal Synaptic Plasticity

Apart from broad changes in TrkB expression in certain brain regions, the subcellular distribution of TrkB, in particular of striatal neurons, appears dysregulated in PD. It has been shown that TrkB is rapidly recruited from intracellular stores to the cell surface of cultured retinal ganglion cells, spinal motor neurons, or hippocampal neurons after depolarization, Ca^2+^ influx, and elevation of cyclic adenosine monophosphate (cAMP) [175,176]. This recruitment to the cell surface is required for the activation and subsequent phosphorylation of TrkB by BDNF [177]. Interestingly, in the 6-OHDA MFB mouse model, L-DOPA administration increases TrkB phosphorylation, specifically in the ipsilateral striatum [169]. Cellular cAMP levels are controlled by the action of adenylyl cyclase (AC), which transforms AMP into cAMP upon its activation [178]. Interestingly, AC can be activated by DRD1 [179] and inhibited by DRD2 [180], which are selectively expressed in either striatal SPNs of the direct pathway (dSPNs) or of the indirect pathway (iSPNs), respectively [181,182,183,184]. Indeed, DRD1 activation leads to the surface translocation of TrkB in cultured striatal neurons [170,185], whereas DRD2 activation decreases TrkB surface levels [186].

**Figure 3:**
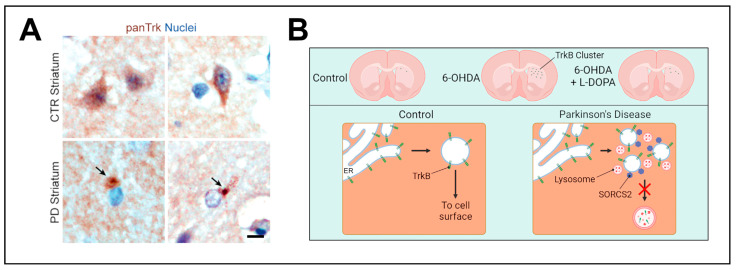
The subcellular distribution of tropomyosin receptor kinase B (TrkB) is altered in patients with Parkinson’s Disease (PD) and rodent models of PD. (**A**) In the striatum of PD patients, Trk receptors accumulate in perinuclear clusters (arrow), whereas in healthy subjects, Trk appears evenly distributed in the soma and on the cell surface of striatal neurons. Scale bar: 10 µm. (**B**) Striatal intracellular clusters of TrkB are readily observed after 6-Hydroxydopamine (6-OHDA) injection. These clusters can be partially prevented with L-DOPA treatment (top). TrkB trafficking to the cell surface is dependent on vesicular exocytotic transport from the endoplasmic reticulum (ER). In models of PD, TrkB-containing vesicles associate with the cargo receptor SORCS-2 but cannot be transported to the cell surface. As a consequence, TrkB forms the observed aggregates, which fail to be cleared via the lysosomal degradation pathway (bottom). Figure modified from Andreska et al. (2023) [170] (licensed under CC BY 4.0).

In the 6-OHDA rat model, TrkB receptors accumulate as intracellular clusters in dSPNs after nigral dopaminergic denervation. This accumulation is also seen in DRD1 KO mice. The aggregation of TrkB can partially be prevented by the administration of L-DOPA after lesioning with 6-OHDA. Interestingly, these clusters are also observed in SPNs of postmortem striatal tissue of patients with PD and also in midbrain dopaminergic neurons, where they are found in close proximity to α-Synuclein-positive Lewy bodies. These TrkB clusters appear to be insoluble and were found to be associated with proteins involved in cell-surface transport and lysosomal degradation pathways in close proximity to the endoplasmic reticulum (ER) (Figure 3) [170]. This raises the question of how TrkB surface transport is regulated and which structures are involved in this process. The current hypothesis is that TrkB accumulates after exiting the ER, as DA depletion leads to a lack of cell-surface transport-promoting signals, and after time, these accumulations form insoluble aggregates that cannot be resolved by the lysosomal machinery. Interestingly, the ER-resident protein Calnexin is involved in the transport of TrkB to the cell surface, as Calnexin KO leads to the attenuation of TrkB transport in cortical precursor cells [187]. It appears that Calnexin acts as a mediator for routing TrkB either to degradation via ER-phagy or to cell surface transport, depending on extracellular signals. Upon Calnexin KO, both are lost, and TrkB accumulates in structures close to the ER, similar to the clusters observed with DA depletion but smaller in size [187]. This makes ER export and ER-phagy important regulators of TrkB processing and consequent cell surface transport.

**Figure 4:**
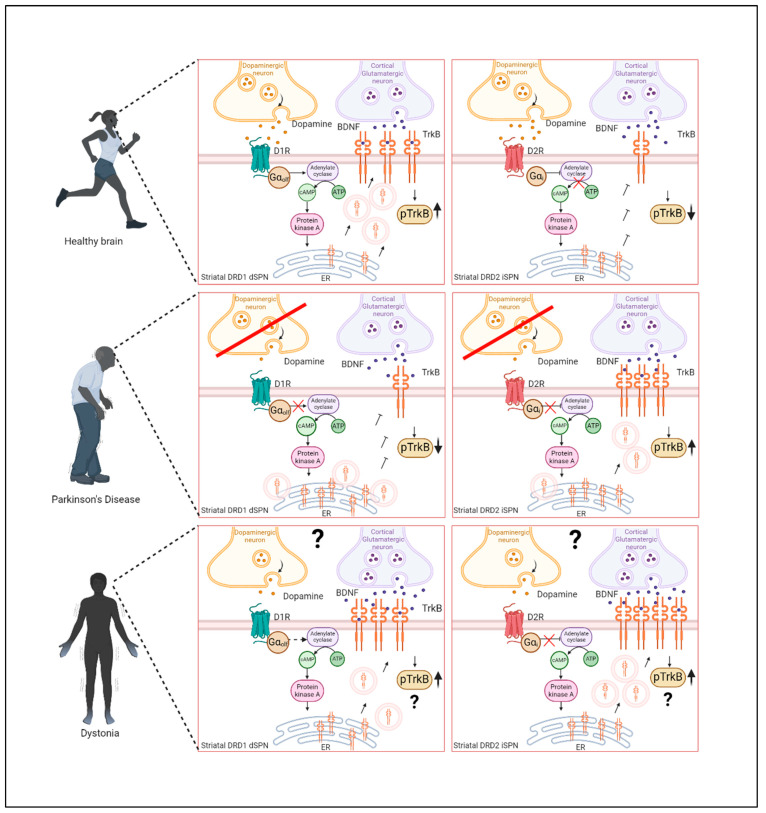
Dopaminergic modulation of the BDNF/TrkB signaling pathways in PD and dystonia. Spiny projection neurons from the direct and indirect pathways in the striatum express D1 and D2 receptors, respectively. D1 receptors recruit Gα_s/olf_ proteins and activate adenylyl cyclase. Increased production of cyclic adenosine monophosphate (cAMP) promotes the translocation of TrkB receptors from intracellular compartments to the cell surface, thus increasing the sensitivity for BDNF and, in turn, increasing the TrkB phosphorylation levels (upwards arrow). Inversely, D2 receptors recruit Gα_i/o/z_ proteins for inhibition of adenylyl cyclase and decreased cAMP production. This inhibits TrkB translocation to the cell surface and decreases its phosphorylation (downwards arrow). In PD, dopaminergic denervation (red slash) causes aberrant TrkB cellular distribution. In dystonia, reduced activation of D2 receptors and enhanced activation of D1 receptors could cause hyperactivation of BDNF/TrkB signaling (question marks indicate hypothetical mechanisms).

Impaired expression of synaptic plasticity is hypothesized to underlie the defective striatal signaling in PD and PD symptom development [115,188,189]. Given the involvement of BDNF/TrkB signaling in synaptic plasticity (see above), the alterations of either BDNF or TrkB in PD have implications for their involvement in the plasticity defects that were found in PD (Figure 4). Abnormalities of synaptic plasticity in models of PD with the involvement of DRD1 and DRD2 have been extensively studied [190,191,192,193,194], but so far, no direct causative role of plasticity defects through BDNF/TrkB abnormalities in PD have been reported.

Taken together, these data underline the importance of BDNF/TrkB signaling for the development and survival of nigrostriatal neurons, as well as for the function of SPNs in health and neurodegenerative diseases.

## 7. BDNF/TrkB Signaling in Dystonia

Dystonia is one of the most prevalent movement disorders after PD, tremor, and restless leg syndrome [195]. It is characterized by involuntary excessive and sustained muscle activity producing abnormal movements, muscle contractions, and postures. It can be focal, involving only cranial, cervical, or limb muscles, or generalized, involving the whole body. Subtypes of focal dystonia include writer’s cramp or musician’s dystonia, which appear after excessive training or repetitive movements of the hands or the body parts involved in the task [196,197]. Symptom onset can range from early childhood to late adulthood, depending on the type of dystonia [198]. There are two main types of dystonia: Sporadic dystonia (idiopathic) and genetic dystonia, which involves over 15 different identified forms [199,200]. Dystonic symptoms can also appear after long-term treatment with Levodopa, one of the most used pharmacological therapies for PD, as part of Levodova-induced dyskinesia (LID) [201].

While the exact mechanisms underlying dystonia are not fully understood, growing evidence suggests that dysfunction of the basal ganglia, including the striatum, plays a significant role, and this dysfunction might be linked to dysregulated neuroplasticity [199]. Quartarone et al. (2006) and other investigators suggest that two factors underlie the pathophysiology of dystonia: genetic predisposition that comes together with activity-dependent environmental factors like peripheral injury or repetitive training and abnormal mechanisms of plasticity [202]. They suggest that the abnormal plasticity in dystonia derives from changes in the plasticity of reflexes and changes in the organization of the cortex. For example, Blepharospasm patients who exhibit involuntary contractions of the periocular musculature demonstrate an enhanced ability to potentiate the trigeminal blink reflex. Although the neural substrates for this abnormal reflex plasticity are unknown, this correlation might indicate that the plasticity mechanisms between the cortex, the striatum, and other areas of the brain could be disrupted [203]. The activity-dependent factors and the abnormal plasticity mechanisms may not be two different sources of the pathogenesis in dystonia, but rather the joint result of activity-dependent upregulation of plasticity-related molecules such as BDNF. One example is dystonia as a result of LID, which is a consequence of maladaptive plasticity in the DA-depleted striatum [204]. The BDNF/TrkB signaling pathway is one of the pathways altered after L-DOPA treatment in rodent models of PD [205], which further indicates that dystonia implicates dysregulation of signaling pathways for motor control and plasticity.

### 7.1. Corticostriatal Plasticity Impairments in Dystonia

A mutation in the DYT-TOR1A (DYT1) gene causes dominantly inherited childhood-onset primary dystonia. Only 30 to 40% of the mutation carriers will develop symptoms. Edwards and colleague’s (2006) main hypothesis was that abnormalities in brain plasticity underlie the pathophysiology of primary DYT-TOR1A dystonia [206]. For this, they recruited four different groups: DYT-TOR1A gene carriers with dystonia, DYT-TOR1A gene carriers without dystonia, patients with sporadic primary dystonia, and healthy control subjects and applied a plasticity probing protocol via repetitive transcranial magnetic stimulation (rTMS) delivered to the motor cortex. They concluded that DYT-TOR1A gene carriers with dystonia and subjects with sporadic dystonia had a significantly prolonged response to rTMS in comparison with healthy subjects. In contrast, DYT-TOR1A gene carriers without dystonia had no significant response to rTMS. These data demonstrate an excessive response to the cortical stimulation in subjects with dystonia but a lack of response in genetically susceptible individuals who have not developed dystonia. Altogether, this suggests that the propensity to undergo plastic change may affect the development of symptoms in genetically vulnerable individuals. This mechanism involves plasticity at corticostriatal synapses in the pathogenesis of primary dystonia. It is possible that these individuals are prone to express higher levels of cortical BDNF, inducing disturbed BDNF/TrkB signaling in the striatum and contributing to the development or progression of dystonia.

Delving into the role of BDNF in DYT-TOR1A dystonia, its childhood onset could indicate changes in plasticity at early development, a period characterized by profuse experience-dependent motor learning. Impairment in striatal plasticity has been demonstrated in different mouse and rat models [207,208]. Maltese et al. (2018), using the Tor1a^+/∆gag^ dystonia mouse model, have shown defects, particularly in corticostriatal plasticity [209]. They reported abnormal functional and structural plasticity in an early developmental stage of SPNs, which was paired with a time-dependent increase in BDNF levels and α-amino-3-hydroxy-5-methyl-4-isoxazolepropionic acid receptor (AMPAR)-mediated currents. Dendritic spine analysis of Tor1a^+/∆gag^ SPNs showed an increase in spine width together with an enhanced AMPA receptor accumulation and a premature start of LTP. The authors found that both pro-BDNF and BDNF levels were significantly higher in Tor1a^+/∆gag^ mice. Consistently, antagonism of BDNF rescued synaptic plasticity deficits and AMPA currents, as BDNF is known to regulate AMPA receptor expression during development. These findings demonstrate that higher levels of BDNF and AMPA currents, together with functional and structural synaptic changes in corticostriatal synapses, are present in this mouse model of dystonia. Thus, abnormal plasticity-related changes in striatal SPNs are part of the pathomechanisms in dystonia [209]. Another study performing cerebellar theta burst stimulation showed a reduction of levodopa-induced dyskinesias together with a decrease in serum BDNF levels, which further provides indirect evidence that an increase in BDNF levels could be associated with dystonic symptoms [210].

### 7.2. Dopamine Signaling Is Involved in Dystonia Pathogenesis

Unlike PD, dystonia is not characterized by a degeneration of the dopaminergic neurons at the SN. However, distinct forms of dystonia involve modified dopaminergic function, including at least four genetically identified forms of dystonia (DYT1, DYT3, DYT5, and DYT11) and tardive dystonia [199]. Postmortem brains of patients with dystonia have shown reduced levels of DA [211]. Likewise, when patients have dystonia after a midbrain stroke, the severity of the symptoms is correlated with the degree of DA denervation in the striatum [212]. Deficiencies in the dopaminergic system may also induce dystonia by changing reflex excitability and this could also impact plasticity.

Changes in striatal dopaminergic signaling have been proposed as part of the pathomechanisms of dystonia [213]. More evidence linking the dopaminergic system with dystonia comes from a DYT-TOR1A postmortem case study, where the SN was found intact, while there was a reduction of DA in the striatum [214]. Another study with three postmortem brains of patients with DYT-TOR1A dystonia showed a decrease in striatal DA and a trend toward a reduction in D1 and D2 receptor activation [215]. Interestingly, some studies show differences between the symptomatically affected and the unaffected mice in a genetic DYT-TOR1A mouse model. The symptomatically affected mutants had decreased striatal DA, while the unaffected mutants exhibited normal or even increased levels of striatal DA [216]. All this indicates that dopaminergic dysregulation might trigger signaling effects that lead to the appearance of motor symptoms, also possibly correlating with the severity of the symptoms.

The effectiveness of L-DOPA in cases of DOPA-responsive dystonia (DRD) has been proven [201,217] and further supports that the pathophysiology of dystonia involves changes in the dopaminergic system. Studies with DRD patients have shown that low levels of levodopa were an effective treatment with long-term benefits [218,219].

### 7.3. BDNF/TrkB Signaling in Dystonia

It has been shown in PD that the sensitivity of SPNs in the striatum to BDNF is differentially regulated by DA. The dopaminergic denervation induces changes in the BDNF/TrkB signaling that also involves aberrant TrkB transport that possibly contributes to the pathomechanisms of the disease [170,186]. In this context, the reduced levels of DA reported in some cases of dystonia could lead to altered TrkB cell surface expression and abnormal BDNF/TrkB signaling in D1-expressing SPNs and D2-expressing SPNs. It would be worth exploring deeper whether this downregulation of DA in dystonia would lead to altered sensitivity to BDNF in neurons from the direct pathway and/or in neurons from the indirect pathway (Figure 4).

Different dystonia types have also shown impaired DA signaling and dysregulations in corticostriatal plasticity. A mouse model of DYT-GNAL (DYT25) dystonia, which involves a mutation in the GNAL gene that encodes for the guanine nucleotide-binding protein subunit Gα_olf_, which is expressed in the striatum and olfactory bulbs, showed impaired DA transmission and motor dysfunction. At the cellular level, downregulated Arc expression was found to increase surface levels of AMPA receptors and loss of D2 receptor-dependent corticostriatal LTD in Gnal^+/−^ rats [220]. In the striatum, dopaminergic activation of D2 SPNs is necessary to induce LTD. In the absence of DA, LTD induction is lost in these neurons [221]. This indicates that D2 SPNs could also be involved in the altered plasticity changes associated with dystonia. The increased surface levels of AMPA receptors found in this study also indicate that the alterations in striatal plasticity might be in part also attributed to changes in LTP occurring in D1 SPNs. Activation of AMPA and NMDA receptors is also integral for plasticity and synaptic transmission at postsynaptic membranes, which is necessary for LTP [89,182]. Another study also found a propensity to generate LTP in corticostriatal slices in the dt^sz^ mutant, a hamster model of paroxysmal dystonia [222]. Using this same model, the corticostriatal synaptic transmission showed significantly higher excitability, which was reflected in enhanced LTP formation in slices of dtsz hamsters in comparison to controls [223]. This could also indicate that the changes in LTP and LTD are associated with changes in dopaminergic signaling.

The dopaminergic dysregulation, the higher excitability of corticostriatal synapses that correlate with increased LTP and loss of LTD [206,220,223], and higher BDNF levels found in a mouse model of DYT-TOR1A dystonia [209], indicate alterations in the direct and indirect pathway SPNs, inducing an imbalance in the opposite direction as in PD. In dystonia, this imbalance could rather be associated with hyperactivation of the BDNF/TrkB signaling, subsequently translating into symptoms such as hyperreflexia, rigidity, and tremor. A hypothetical model is illustrated in Figure 4, together with the altered BDNF/TrkB signaling mechanisms in PD.

### 7.4. Effects of Deep Brain Stimulation and Neuroplasticity Modulation in the Therapy of Dystonia

DBS of the GPi shows efficacy for refractory segmental, cervical, and generalized dystonia in children and adults [224,225,226]. The benefits of DBS have proven long-term efficacy [227]. The GPi DBS targets the cortical-basal ganglia–thalamo–cortical motor loop [228]. Due to the largely unknown pathophysiology of dystonia, the precise mechanism by which GPi DBS alleviates dystonia remains unclear. Some studies suggest that GPi DBS enhances output from the targeted nucleus and activates adjacent fiber pathways. This activation leads to a complex interplay of excitatory and inhibitory pathways that influence the entire basal ganglia-thalamocortical network. The stimulation-induced modulation of neuronal activity apparently disrupts the transmission of pathological bursting and oscillatory patterns within the network, thereby improving sensorimotor processing and reducing symptoms of the disease [229]. These effects could also involve altered production and release of BDNF from corticostriatal afferences, and also altered responses if SPNs to BDNF that is provided from cortical or dopaminergic afferences.

Although the evidence of altered BDNF/TrkB signaling in corticostriatal synapses in dystonia is scarce, and more evidence is necessary to further clarify the specific modulatory plasticity mechanisms in dystonia, is it likely that the pathophysiology of many cases of dystonia involves changes in the dopaminergic system that seem to be correlated with plasticity and changes in BDNF signaling, at least in the symptomatologic genetic cases. More experimental evidence is required to find the specific role of BDNF and the possible disturbances either in the motor cortex or the striatum that induce motor symptoms in dystonia.

## 8. Conclusions

This review brings together evidence on the role of BDNF in motor control and neuroprotection that essentially contribute to the pathophysiology of Parkinson’s disease and possibly dystonia. Therapeutic approaches such as deep brain stimulation could mediate their beneficial effects on motor symptoms and neuroprotection through BDNF/TrkB signaling. Therefore, targeting BDNF/TrkB signaling to restore neuroplasticity mechanisms at corticostriatal synapses could open potential therapeutic avenues for treating these neurological diseases. Additionally, this approach may benefit other neurodegenerative conditions or neuropsychiatric disorders involving altered BDNF levels, such as Huntington’s disease, Alzheimer’s disease, and schizophrenia.

## Data Availability

Dataset available on request from the authors.
